# The effectiveness of booster vaccination of inactivated COVID-19 vaccines against susceptibility, infectiousness, and transmission of omicron BA.2 variant: a retrospective cohort study in Shenzhen, China

**DOI:** 10.3389/fimmu.2024.1359380

**Published:** 2024-05-31

**Authors:** Yuxue Liao, Jiao Su, Jieru Zhao, Zhen Qin, Zhuo’Ao Zhang, Wei Gao, Jia Wan, Yi Liao, Xuan Zou, Xiaofeng He

**Affiliations:** ^1^ Office of Emergency, Shenzhen Center for Disease Control and Prevention, Shenzhen, China; ^2^ Department of Biochemistry, Changzhi Medical College, Changzhi, China; ^3^ Department of Infectious Disease, Heping Hospital Affiliated to Changzhi Medical College, Changzhi, China; ^4^ Class of 2002 of the Department of Preventive Medicine, Changzhi Medical College, Changzhi, China; ^5^ Institute of Evidence-Based Medicine, Heping Hospital Affiliated to Changzhi Medical College, Changzhi, China

**Keywords:** COVID-19, vaccine effectiveness, BA.2 subvariant, susceptibility, infectiousness, transmission

## Abstract

Little studies evaluated the effectiveness of booster vaccination of inactivated COVID-19 vaccines against being infected (susceptibility), infecting others (infectiousness), and spreading the disease from one to another (transmission). Therefore, we conducted a retrospective cohort study to evaluate the effectiveness of booster vaccination of inactivated COVID-19 vaccines against susceptibility, infectiousness, and transmission in Shenzhen during an Omicron BA.2 outbreak period from 1 February to 21 April 2022. The eligible individuals were classified as four sub-cohorts according to the inactivated COVID-19 vaccination status of both the close contacts and their index cases: group 2-2, fully vaccinated close contacts seeded by fully vaccinated index cases (reference group); group 2-3, booster-vaccinated close contacts seeded by fully vaccinated index cases; group 3-2, fully vaccinated close contacts seeded by booster-vaccinated index cases; and group 3-3, booster-vaccinated close contacts seeded by booster-vaccinated index cases. Univariate and multivariate logistic regression analyses were applied to estimate the effectiveness of booster vaccination. The sample sizes of groups 2-2, 2-3, 3-2, and 3-3 were 846, 1,115, 1,210, and 2,417, respectively. We found that booster vaccination had an effectiveness against infectiousness of 44.9% (95% CI: 19.7%, 62.2%) for the adults ≥ 18 years, 62.2% (95% CI: 32.0%, 78.9%) for the female close contacts, and 60.8% (95% CI: 38.5%, 75.1%) for the non-household close contacts. Moreover, booster vaccination had an effectiveness against transmission of 29.0% (95% CI: 3.2%, 47.9%) for the adults ≥ 18 years, 38.9% (95% CI: 3.3%, 61.3%) for the female close contacts, and 45.8% (95% CI: 22.1%, 62.3%) for the non-household close contacts. However, booster vaccination against susceptibility did not provide any protective effect. In summary, this study confirm that booster vaccination of the inactivated COVID-19 vaccines provides low level of protection and moderate level of protection against Omicron BA.2 transmission and infectiousness, respectively. However, booster vaccination does not provide any protection against Omicron BA.2 susceptibility.

## Introduction

In December 2019, an emerging infectious disease named coronavirus disease 2019 (COVID-19) caused by severe acute respiratory syndrome (SARS-CoV-2) virus was first found in Wuhan, China, and then, this virus is widespread all over the world and it has existed for nearly four years (1–3). Moreover, SARS-CoV-2 is constantly undergoing various variants, which have been categorized as variants of concern, variants of interest, and variants under monitoring by World Health Organization (WHO) (4). Variants of concern mainly involved Alpha, Beta, Gamma, Delta, and Omicron (4). The Omicron (B.1.1.529) variant was first identified in South Africa on November 24, 2021 and it quickly removed of all other strains and became the dominant strain worldwide because it had stronger transmissibility and immune escape ability (5–8).

COVID-19 vaccination is still regarded as one of the most effective methods for achieving herd immunity to control the disease and as an indispensable part of the long-term management of SARS-CoV-2 pandemic (9–12). COVID-19 vaccines mainly included inactivated virus vaccines (CoronaVac, WIV04, HB02, and BBV152), protein subunit vaccines (NVX-CoV2373 and SCB-2019), RNA-based vaccines (BNT162b2 and mRNA-1273), and Viral vector (non-replicating) vaccines (AZD1222 [ChAdOx1 nCoV-19], Sputnik V, Ad26.COV2.S, and Ad5-nCoV) (13). Large-scale phase III randomized clinical trials (RCTs) indicated that the full vaccination of the above COVID-19 vaccines had good protective effect against symptomatic COVID-19, especially severe COVID-19, COVID-19-related hospitalization, and COVID-19-related death (14–23). However, the results of RCTs concerning the COVID-19 vaccines efficacy were reported before the pandemic of the Omicron variant. It is an essential complement applying real-world data to evaluate the COVID-19 vaccines effectiveness (VE) because real-world studies have a larger and wider range of population and consider various background factors beyond the experimental conditions (24–28).

Although primary series of COVID-19 vaccines had adequate protective effect against symptomatic COVID-19, especially severe COVID-19 (13). However, the VE was rapidly waning over time (29). Therefore, many countries launched a campaign of booster vaccination by applying either homologous or heterologous COVID-19 vaccines. Booster vaccination was usually administered as a third dose when at least five-six months after full vaccination of COVID-19 vaccines. Currently, most real-world studies mainly assessed the effectiveness of COVID-19 booster vaccination of two mRNA vaccines against different outcomes of Omicron infection (13). In mainland China, since early 2021, two inactivated COVID-19 vaccines (HB02 [Sinopharm] and CoronaVac [Sinovac]) were most frequently used. Although several studies have assessed the effectiveness of booster vaccination of inactivated COVID-19 vaccines made in China against different outcomes of Omicron BA.2 infection (30–35). However, their studies (30–35) have only focused on the VE in preventing being infected or severe COVID-19. To our knowledge, evaluating the effectiveness of a vaccine includes both measure indicators: preventing infection and preventing infecting others. VE in preventing infection is the most common and basic indicator. In this study, this measure indicator is specifically defined as VE against susceptibility, which refers to the extent to which the vaccine decreasing secondary infection. It is a new concept on the VE in preventing infecting others, which has only attracted attention in recent years (36–38). VE against infectiousness was also named as VE against infectivity in some studies and may be interpreted as the reduction in transmission risk from a primary to secondary infection, which refers to the extent to which the vaccine reduces infectivity among individuals already infected. Moreover, vaccination can prevent transmission by providing protection against infection and simultaneously decreasing the infectivity of vaccinated individuals who become infected. Therefore, VE against transmission is defined as the combination of VE against susceptibility and VE against infectiousness in this study, which refers to the vaccine’s ability to break the chain of virus transmission and interrupt community spread.

We conducted a retrospective cohort study to assess the effectiveness of booster vaccination of inactivated COVID-19 vaccines against be infected (susceptibility), infecting others (infectiousness), and disease transmission among individuals (transmission) in Shenzhen during an Omicron BA.2 outbreak period from 1 February to 21 April 2022. Of note, the inactivated COVID-19 vaccines used were the initial SARS-CoV-2 strain vaccines in this study.

## Methods

### Study setting

This article was reported applying the S1 STROBE Checklist. With an Omicron BA.2 sub-lineage outbreak background in Shenzhen, Guangdong, China, reported the first COVID-19 case on February 1, 2022 and reached a peak of infections on March 15, 2022. On the basis of the “zero COVID-19” policy, the local government quickly took a series of control measures mainly including massive nucleic acid testing, contact tracing, isolation of COVID-19 cases, and quarantine of close contacts. Therefore, this major epidemic was controlled on April 21, 2022.

### Study design and participants

This retrospective cohort study was conducted to analyze the close contacts of all confirmed COVID-19 cases by reverse transcription polymerase chain reaction (RT-PCR) tests in Shenzhen, China from February 1 to April 21, 2022. Close contacts referred to the individuals who were in the same exposure settings within close proximity without any effective protection as COVID-19 cases or SARS-CoV-2 positive asymptomatic infections two days before. In this study, individuals at risk of exposure were regarded as close contacts of confirmed cases. Contact tracing measures were conducted by Shenzhen Center for Disease Control and Prevention allows for matching the close contacts with their index cases. All the close contacts were traced, compulsorily quarantined, and tested every 2-3 days by RT-PCR tests for SARS-CoV-2 to monitor whether they were infected with SARS-CoV-2. All the secondary SARS-CoV-2 infections were the close contacts of their index cases before they became infected. Notably, all the secondary SARS-CoV-2 infections and uninfected close contacts made up a cohort together. For individuals with BA.2 sub-lineage infection, we extracted data, which mainly included age, sex, history of exposure, contact setting (i.e., household and non- household settings), onset date of clinical symptoms, first positive test date, and history of COVID-19 vaccination. Those close contacts who eventually tested positive for COVID-19 were treated as infected individuals (infectees) and their index cases (who were originally confirmed to have COVID-19) as infectors. Of note, close contacts became index cases when testing positive, so this study may include some individuals both as close contacts and as index cases. Furthermore, the matching involved creating multiple pairs of index-exposed individuals in this study. Overall, 8466 close contacts were matched with 644 index cases in the present study. Then, close contacts were excluded if both the close contacts and their index cases received non-inactivated COVID-19 vaccines or aged 0-17 years. Moreover, close contacts were excluded if both the close contacts and their index cases were unvaccinated, received partial vaccination, and those who were infected or exposed within 14 days of their last vaccination. The eligible individuals were classified as four sub-cohorts according to the inactivated COVID-19 vaccination status of both the close contacts and their index cases: fully vaccinated close contacts seeded by fully vaccinated index cases (group 2-2 [reference group]), booster-vaccinated close contacts seeded by fully vaccinated index cases (group 2-3), fully vaccinated close contacts seeded by booster-vaccinated index cases (group 3-2), and booster-vaccinated close contacts seeded by booster-vaccinated index cases (group 3-3).

### Vaccination status and outcome

In this study, full vaccination and booster vaccination were defined as ≥ 14 days after second-dose and third-dose vaccination of inactivated COVID-19 vaccines, respectively. The primary outcome was Omicron BA.2 infection within close contacts.

### Statistical analyses

Baseline characteristics were analyzed using the number (%) for categorical data. Differences in proportions were analyzed with the chi-squared test. The secondary attack rate was calculated by dividing the number of individuals with secondary infections by the overall number of close contacts related to index cases.

In this study, we applied the odds ratio (OR) to evaluate the association between the inactivated COVID-19 vaccination status (i.e., four sub-cohorts) and the risk of Omicron BA.2 secondary infection. The crude OR was calculated by applying a univariate logistic regression analysis, and the adjusted OR (aOR) was calculated using a multivariable ordinary logistic regression analysis by adjusting for potential confounding variables, including sex and age of close contacts and their index cases, contact settings, and exposure to index cases before or after symptom onset of the indexes. The crude or adjusted VE (aVE) was calculated as (1−OR) × 100%, and the OR is the odds ratio for the rate of secondary infection. In this study, the VE of booster vaccination of inactivated COVID-19 vaccines against susceptibility, infectiousness, and transmission were calculated through group 2-3 versus group 2-2, group 3-2 versus group 2-2, and group 3-3 versus group 2-2, respectively. Moreover, subgroup analyses were conducted according to sex and contact setting. All statistical analyses were conducted applying IBM SPSS Statistics 25.0.

## Results

### General information of the close contacts

**Figure 1:**
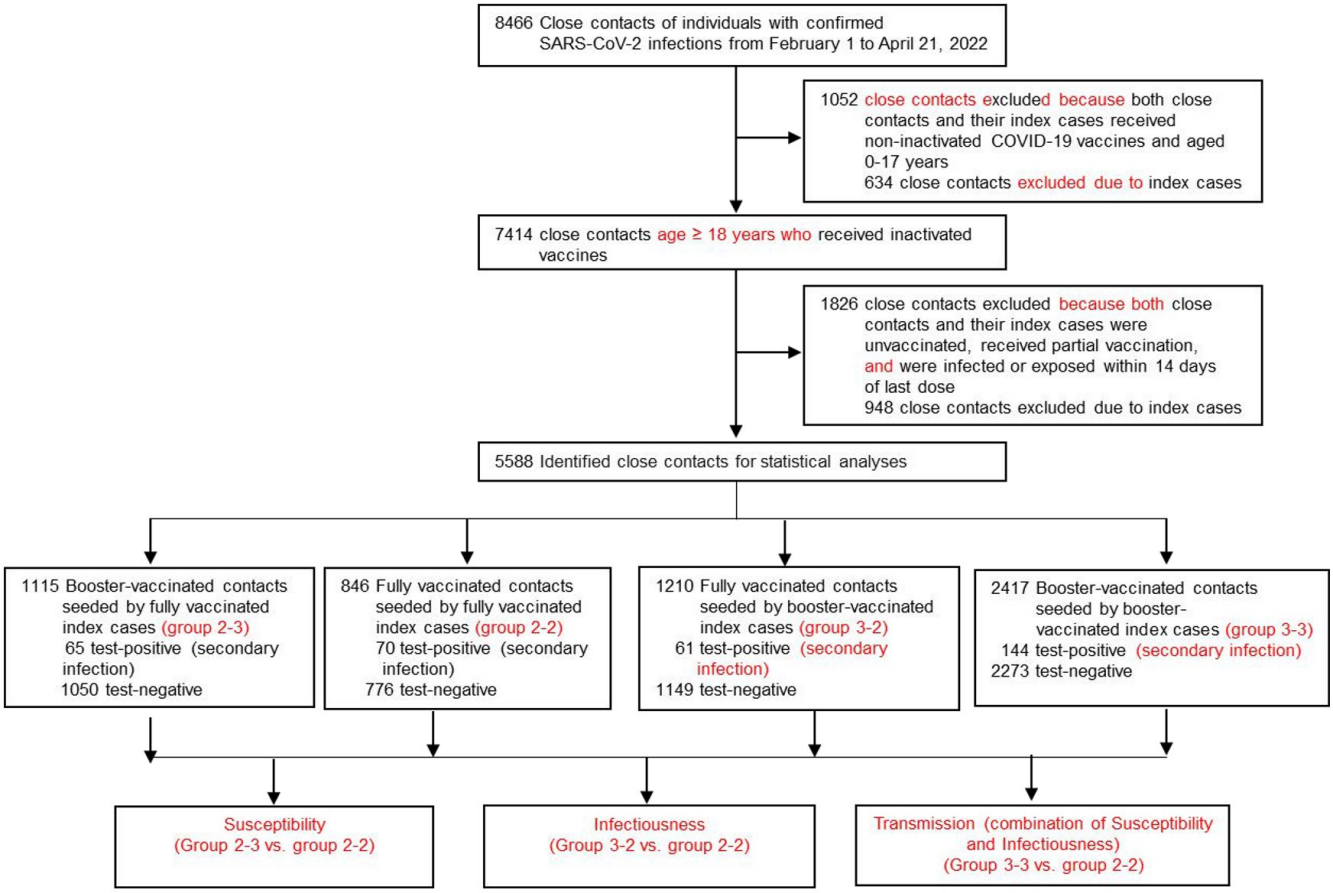
Flow chart of sample selection and grouping.

8,466 close contacts associated with index cases were identified from February 1 to April 21, 2022. Among them, 1,052 individuals were excluded because both these close contacts and their index cases received non-inactivated COVID-19 vaccines or aged 0-17 years. Moreover, we excluded 1,826 individuals who were unvaccinated, received partial vaccination, or were infected or exposed within 14 days of last dose. Therefore, 5,588 close contacts were included to analyze the effectiveness of booster vaccination of inactivated COVID-19 vaccines against susceptibility, infectiousness, and transmission (**Figure 1**).

According to the inactivated COVID-19 vaccination status of both the close contacts and their index cases, the close contacts cohort was classified as four subgroups: group 2-2, group 2-3, group 3-2, and group 3-3, as stated earlier. The sample sizes of groups 2-2, 2-3, 3-2, and 3-3 were 846, 1,115, 1,210, and 2,417, respectively (**Figure 1**, **Table 1**), respectively. Among the 5,588 close contacts, 3,472 (62.1%) were male and 5,372 (96.1%) individuals aged 18-59 years old (**Table 1**). Moreover, for index cases, 3,481 (62.3%) were male and 5,434 (97.2%) individuals aged 18-59 years old (**Table 1**). In terms of contact setting, there were 888 (15.9%) and 4700 (84.1%) household and non-household settings (**Table 1**). Moreover, there were 2,605 (46.6%) and 2,983 (53.1%) individuals with exposure to index cases after or before onset of indexes, respectively, (**Table 1**). Furthermore, there were significantly different (*P* < 0.001) for age, age and gender of index cases, and contact settings among the four sub-cohorts (**Table 1**).

**Table 1:** Baseline characteristics of close contacts of Omicron BA.2 infections who received full vaccination and booster vaccination.

Characteristics	Contacts seeded by fully vaccinated cases, n (column, %)		Contacts seeded by booster-vaccinated cases, n (column, %)		Overall close contacts (5588)	*P value*
	Group 2-2: contacts of full vaccination (n = 846 [15.1])	Group 2-3: contacts of booster vaccination (n = 1115 [19.9])	Group 3-2: contacts of full vaccination (n = 1210 [21.7])	Group 3-3: contacts of booster vaccination (n = 2417 [43.3])		
Age (years)						
18-59	804 (95.0)	1096 (98.3)	1116 (92.2)	2356 (97.5)	5372 (96.1)	< 0.001
≥ 60	42 (5.0)	19 (1.7)	94 (7.8)	61 (2.5)	216 (3.9)
Age of index cases (years)						
18-59	800 (94.6)	1062 (95.2)	1194 (98.7)	2378 (98.4)	5434 (97.2)	< 0.001
≥ 60	46 (5.4)	53 (4.8)	16 (1.3)	39 (1.6)	154 (2.8)
Gender						
Male	514 (60.8)	710 (63.7)	750 (62.0)	1498 (62.0)	3472 (62.1)	0.605
Female	332 (39.2)	405 (36.3)	460 (38.0)	919 (38.0)	2116 (37.9)
Gender of index cases						
Male	536 (63.4)	813 (72.9)	733 (60.6)	1399 (57.9)	3481 (62.3)	< 0.001
Female	310 (36.6)	302 (27.1)	477 (39.4)	1018 (42.1)	2107 (37.7)
Contact setting						
Household	170 (20.1)	235 (21.1)	159 (12.8)	324 (13.4)	888 (15.9)	< 0.001
Non-household	676 (79.9)	880 (78.9)	1051 (87.2)	2093 (86.6)	4700 (84.1)
Exposure to index cases at onset *						
Yes	379 (44.8)	495 (44.4)	579 (47.9)	1152 (47.7)	2605 (46.6)	0.162
No	467 (55.2)	620 (55.6)	631 (52.1)	1265 (52.3)	2983 (53.1)

^*^Close contacts were exposed to index cases at the time of symptom onset of the indexes.

### Effectiveness of booster vaccination of inactivated COVID-19 vaccines against susceptibility, infectiousness, and transmission

**Figure 2:**
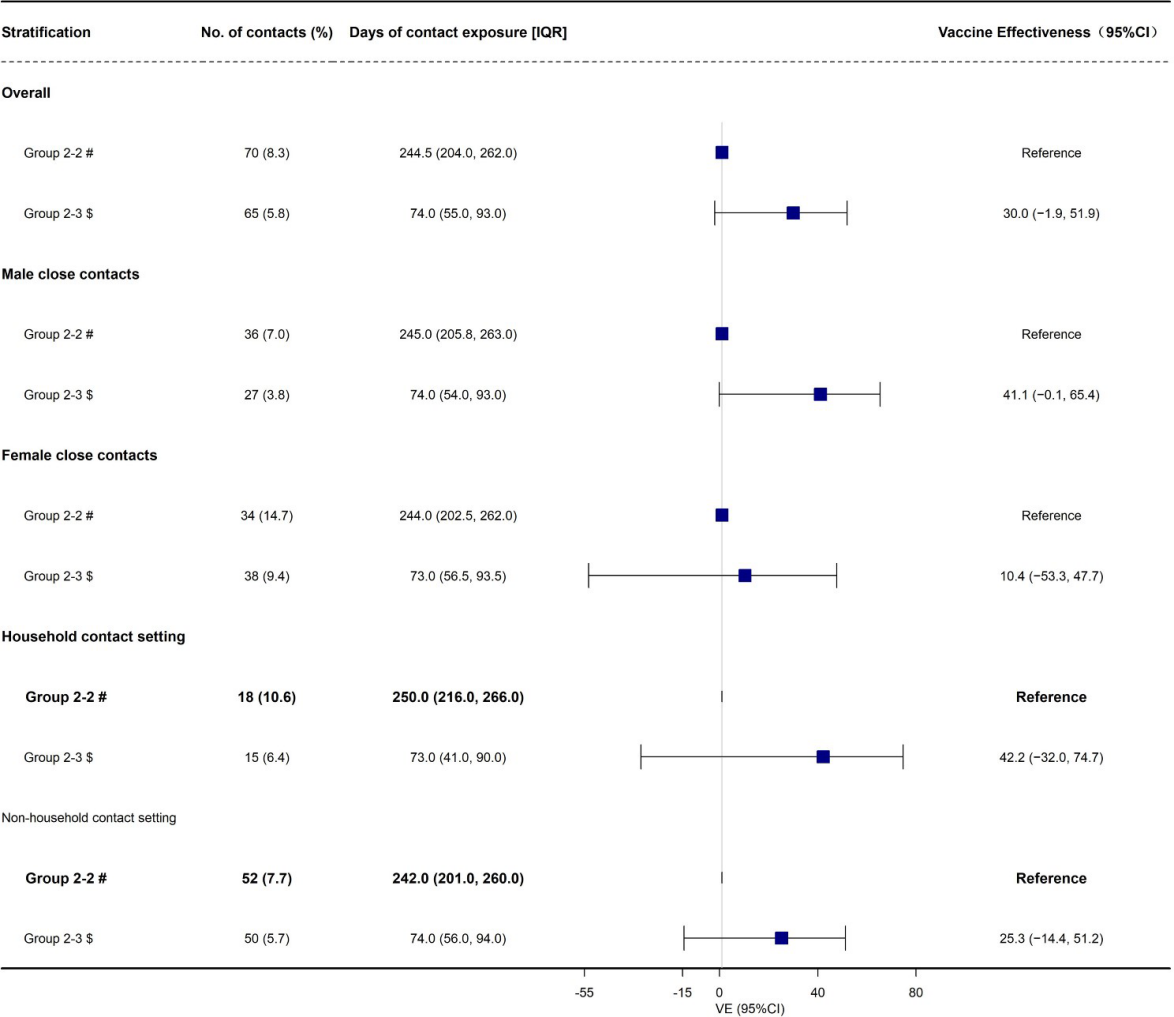
The effectiveness of booster Vaccination of inactivated COVID-19 vaccines against be infected (susceptibility) duo to Omicron BA.2 variant, in Shenzhen, China VE, vaccine effectiveness. # Fully vaccinated close contacts seeded by fully vaccinated index cases; $ Booster-vaccinated close contacts seeded by fully vaccinated index cases.

The overall effectiveness of booster vaccination of inactivated COVID-19 vaccines against susceptibility of Omicron BA.2 variant was 30.0% (95% CI: −1.9%, 51.9%, **Table 2**, **Figure 2**) and did not provide any protective effect. Moreover, we found that booster vaccination of inactivated COVID-19 vaccines against susceptibility also did not provide any protective effect in the subgroups according to gender and contact setting (**Table 2**, **Figure 2**).

**Table 2:** The effectiveness of booster Vaccination of inactivated COVID-19 vaccines against susceptibility of Omicron BA.2 variant, in Shenzhen, China.

Stratification	Number of contacts (%)		Time from last vaccine to contact exposure, days [IQR]			Crude			Adjusted *			
	Test-positive	Test-negative	For contacts	For index cases		OR (95% CI)		VE (95% CI), %	OR (95% CI)		VE (95% CI), %	
Overall												
Group 2-2 ^#^	70 (8.3)	776 (91.7)	244.5 (204.0, 262.0)	249.2 (216.0, 263.0)		Ref.		..	Ref.		..
Group 2-3 ^$^	65 (5.8)	1050 (94.2)	74.0 (55.0, 93.0)	250.0 (210.0, 260.0)		0.686 (0.484, 0.974)		31.4 (2.6, 51.6)	0.700 (0.481, 1.019)		30.0 (−1.9, 51.9)
Male close contacts												
Group 2-2 ^#^	36 (7.0)	478 (93.0)	245.0 (205.8, 263.0)	250.0 (216.0, 262.0)		Ref.		..	Ref.		..
Group 2-3 ^$^	27 (3.8)	683 (96.2)	74.0 (54.0, 93.0)	250.0 (211.0, 257.0)		0.525 (0.314, 0.876)		47.5 (12.4, 68.6)	0.589 (0.346, 1.001)		41.1 (−0.1, 65.4)
Female close contacts												
Group 2-2 ^#^	34 (14.7)	298 (85.3)	244.0 (202.5, 262.0)	248.5 (215.5, 263.0)		Ref.		..	Ref.		..
Group 2-3 ^$^	38 (9.4)	367 (90.6)	73.0 (56.5, 93.5)	248.0 (206.0, 263.0)		0.908 (0.557, 1.477)		9.2 (−47.7, 44.3)	0.896 (0.523, 1.533)		10.4 (−53.3, 47.7)
Household contact setting												
Group 2-2 ^#^	18 (10.6)	152 (89.4)	250.0 (216.0, 266.0)	253.0 (246.3, 264.0)		Ref.		..	Ref.		..
Group 2-3 ^$^	15 (6.4)	220 (93.6)	73.0 (41.0, 90.0)	253.0 (249.0, 253.0)		0.576 (0.281, 1.178)		42.4 (−17.8, 71.9)	0.578 (0.253, 1.320)		42.2 (−32.0, 74.7)
Non-household contact setting												
Group 2-2 ^#^	52 (7.7)	624 (92.3)	242.0 (201.0, 260.0)	243.0 (207.8, 263.0)		Ref.		..	Ref.		..
Group 2-3 ^$^	50 (5.7)	830 (94.3)	74.0 (56.0, 94.0)	243.0 (200.0, 261.0)		0.723 (0.484, 1.080)		37.7 (−8.0, 51.6)	0.747 (0.488, 1.144)		25.3 (−14.4, 51.2)

VE, vaccine effectiveness; * Variables adjusted in the model were sex of index cases and their close contacts, age of index cases and their close contacts, contact settings, and exposure to index cases at onset of indexes; ^#^ Fully vaccinated close contacts seeded by fully vaccinated index cases; ^$^ Booster-vaccinated close contacts seeded by fully vaccinated index cases.

**Figure 3:**
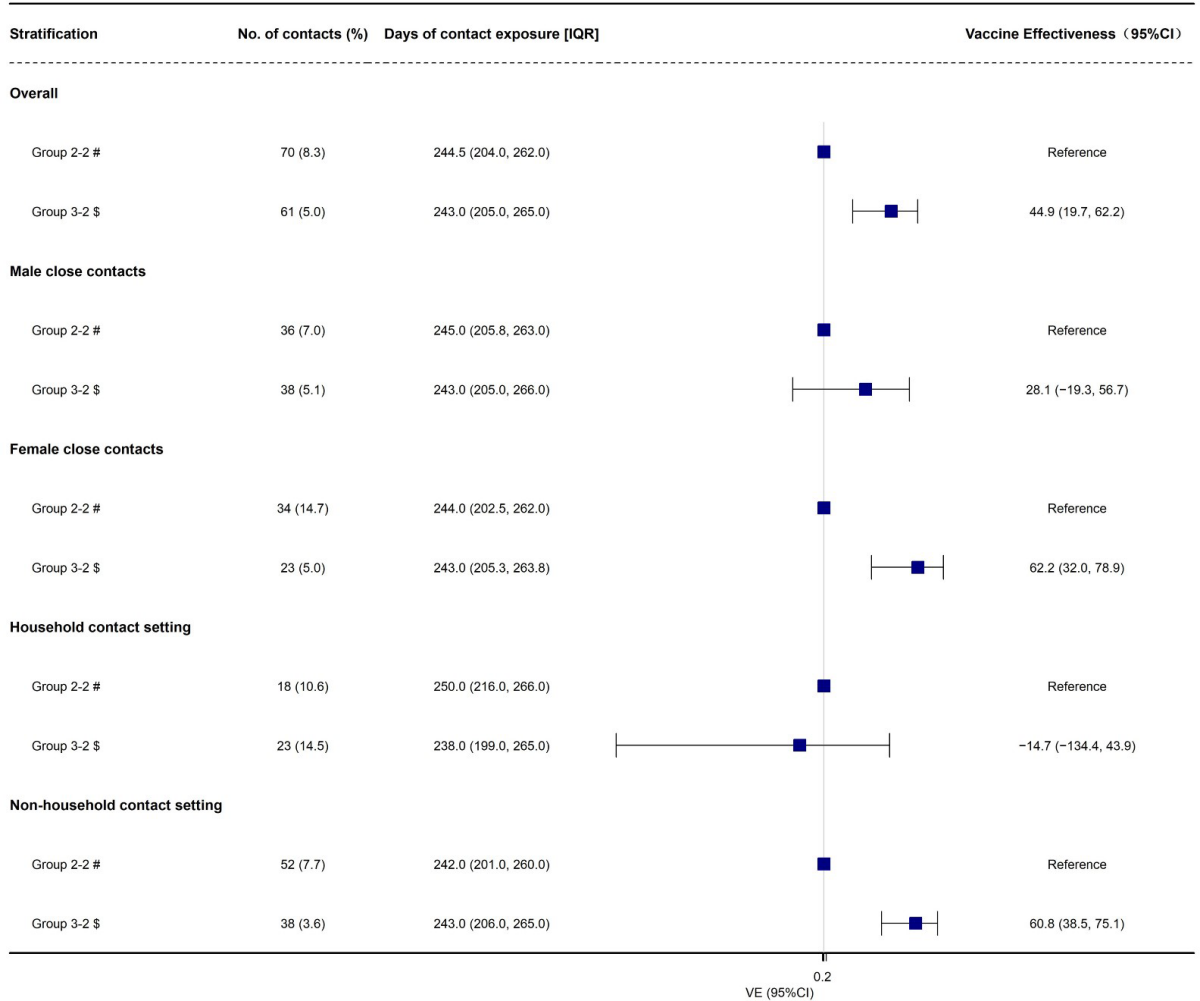
The effectiveness of booster vaccination of inactivated COVID-19 vaccines against infectiousness due to Omicron BA.2 variant, in Shenzhen, China VE, vaccine effectiveness. # Fully vaccinated close contacts seeded by fully vaccinated index cases; $ Fully vaccinated close contacts seeded by booster-vaccinated index cases.

Then, we found that the overall effectiveness of booster vaccination of inactivated COVID-19 vaccines against infectiousness of Omicron BA.2 variant was 44.9% (95% CI: 19.7%, 62.2%, **Table 3**, **Figure 3**). Moreover, we found that the effectiveness of booster vaccination of inactivated COVID-19 vaccines against infectiousness was 62.2% (95% CI: 32.0%, 78.9%) and 60.8% (95% CI: 38.5%, 75.1%) for the female close contacts and the non-household close contacts, respectively (**Table 3**, **Figure 3**). However, booster vaccination of inactivated COVID-19 vaccines against infectiousness did not provide any protective effect for the male close contacts and household close contacts (**Table 3**, **Figure 3**).

**Table 3:** The effectiveness of booster vaccination of inactivated COVID-19 vaccines against infectiousness of Omicron BA.2 variant, in Shenzhen, China.

Stratification	Number of contacts (%)		Time from last vaccine to contact exposure, days [IQR]		Crude		Adjusted *	
	Test-positive	Test-negative	For contacts	For index cases	OR (95% CI)	VE (95% CI), %	OR (95% CI)	VE (95% CI), %
Overall								
Group 2-2 ^#^	70 (8.3)	776 (91.7)	244.5 (204.0, 262.0)	249.2 (216.0, 263.0)	Ref.	..	Ref.	..
Group 3-2 ^$^	61 (5.0)	1149 (95.0)	243.0 (205.0, 265.0)	84.0 (66.0, 105.3)	0.589 (0.413, 0.840)	41.1 (16.0, 58.7)	0.551 (0.378, 0.803)	44.9 (19.7, 62.2)
Male close contacts								
Group 2-2 ^#^	36 (7.0)	478 (93.0)	245.0 (205.8, 263.0)	250.0 (216.0, 262.0)	Ref.	..	Ref.	..
Group 3-2 ^$^	38 (5.1)	712 (94.9)	243.0 (205.0, 266.0)	85.0 (66.0, 103.0)	0.709 (0.443, 1.134)	29.1 (−13.4, 55.7)	0.719 (0.433, 1.193)	28.1 (−19.3, 56.7)
Female close contacts								
Group 2-2 ^#^	34 (14.7)	298 (85.3)	244.0 (202.5, 262.0)	248.5 (215.5, 263.0)	Ref.	..	Ref.	..
Group 3-2 ^$^	23 (5.0)	437 (95.0)	243.0 (205.3, 263.8)	82.0 (63.0, 112.8)	0.461 (0.266, 0.799)	53.9 (20.1, 73.4)	0.378 (0.211, 0.680)	62.2 (32.0, 78.9)
Household contact setting								
Group 2-2 ^#^	18 (10.6)	152 (89.4)	250.0 (216.0, 266.0)	253.0 (246.3, 264.0)	Ref.	..	Ref.	..
Group 3-2 ^$^	23 (14.5)	136 (85.5)	238.0 (199.0, 265.0)	87.0 (69.0, 96.0)	1.428 (0.739, 2.760)	−42.8 (−176.0, 26.1)	1.147 (0.561, 2.344)	−14.7 (−134.4, 43.9)
Non-household contact setting								
Group 2-2 ^#^	52 (7.7)	624 (92.3)	242.0 (201.0, 260.0)	243.0 (207.8, 263.0)	Ref.	..	Ref.	..
Group 3-2 ^$^	38 (3.6)	1013 (96.4)	243.0 (206.0, 265.0)	83.0 (66.0, 110.0)	0.450 (0.293, 0.692)	55.0 (30.8, 70.7)	0.392 (0.249, 0.615)	60.8 (38.5, 75.1)

VE, vaccine effectiveness; * Variables adjusted in the model were sex of index cases and their close contacts, age of index cases and their close contacts, contact setting, and exposure to index cases at onset of indexes; ^#^ Fully vaccinated close contacts seeded by fully vaccinated index cases; ^$^ Fully vaccinated close contacts seeded by booster-vaccinated index cases.

**Figure 4:**
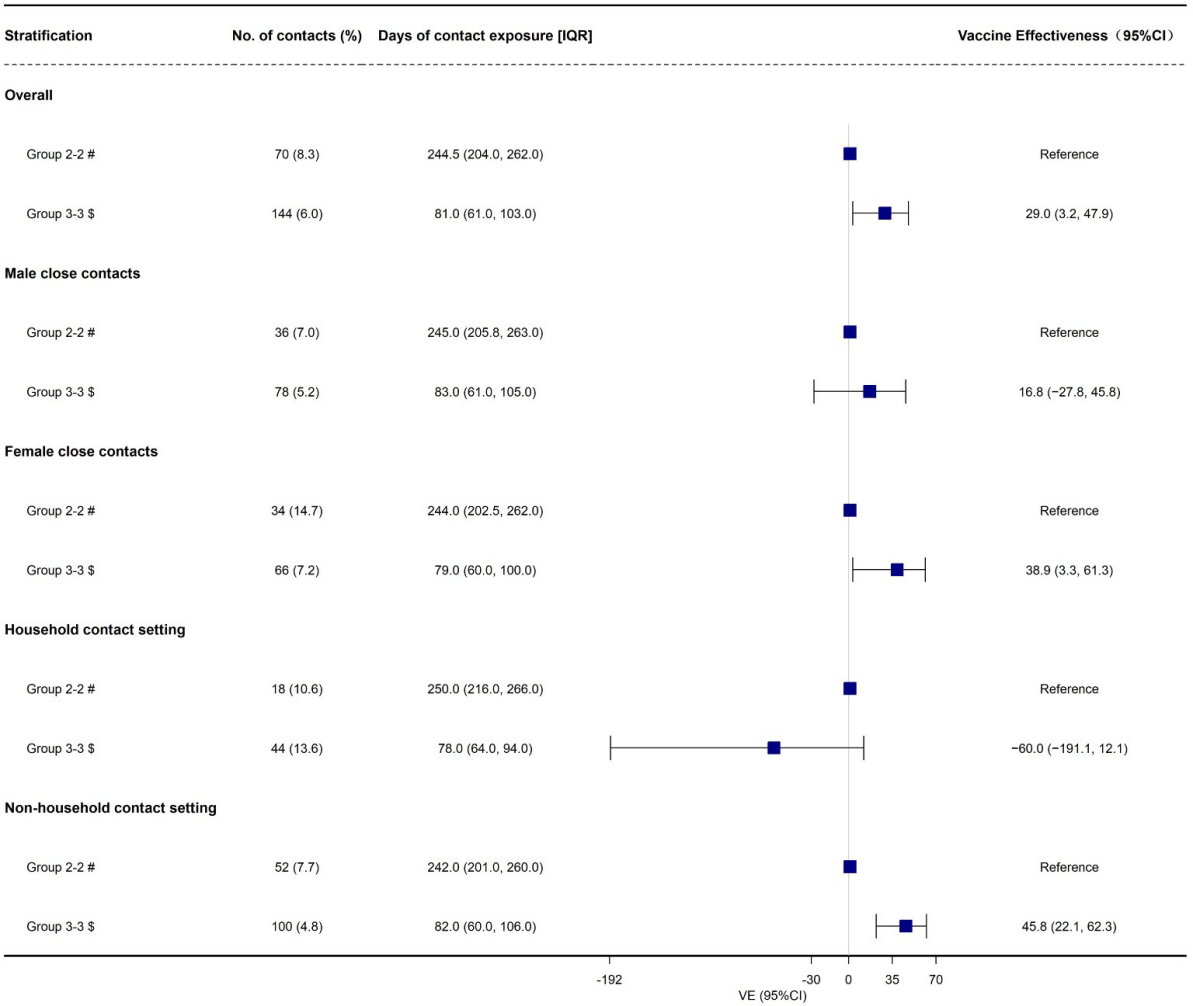
The effectiveness of booster vaccination of inactivated COVID-19 vaccines against transmission of Omicron BA.2, in Shenzhen, China. # Fully vaccinated close contacts seeded by fully vaccinated index cases; $ Booster-vaccinated close contacts seeded by booster-vaccinated index cases.

Finally, we found that the overall effectiveness of booster vaccination of inactivated COVID-19 vaccines against transmission of Omicron BA.2 variant was 29.0% (95% CI: 3.2%, 47.9%, **Table 4**, **Figure 4**). Moreover, we found that the effectiveness of booster vaccination of inactivated COVID-19 vaccines against transmission was 38.9% (95% CI: 3.3%, 61.3%) and 45.8% (95% CI: 22.1%, 62.3%) for the female close contacts and the non-household close contacts, respectively (**Table 4**, **Figure 4**). However, booster vaccination of inactivated COVID-19 vaccines against transmission did not provide any protective effect for the male close contacts and household close contacts (**Table 4**, **Figure 4**).

**Table 4:** The Effectiveness of booster vaccination of Inactivated COVID-19 Vaccines Against transmission of Omicron BA.2, in Shenzhen, China.

Stratification	Number of contacts (%)		Time from last vaccine to contact exposure, days [IQR]		Crude		Adjusted *	
	Test-positive	Test-negative	For contacts	For index cases	OR (95% CI)	VE (95% CI), %	OR (95% CI)	VE (95% CI), %
Overall								
Group 2-2^#^	70 (8.3)	776 (91.7)	244.5 (204.0, 262.0)	249.2 (216.0, 263.0)	Ref.	..	Ref.	..
Group 3-3 ^$^	144 (6.0)	2273 (94.0)	81.0 (61.0, 103.0)	88.0 (74.0, 106.5)	0.702 (0.522, 0.945)	29.8 (5.5, 47.8)	0.710 (0.521, 0.968)	29.0 (3.2, 47.9)
Male close contacts								
Group 2-2^#^	36 (7.0)	478 (93.0)	245.0 (205.8, 263.0)	250.0 (216.0, 262.0)	Ref.	..	Ref.	..
Group 3-3 ^$^	78 (5.2)	1420 (94.8)	83.0 (61.0, 105.0)	88.0 (766.0, 105.0)	0.729 (0.485, 1.097)	27.1 (−9.7, 51.5)	0.832 (0.542, 1.278)	16.8 (−27.8, 45.8)
Female close contacts								
Group 2-2^#^	34 (14.7)	298 (85.3)	244.0 (202.5, 262.0)	248.5 (215.5, 263.0)	Ref.	..	Ref.	..
Group 3-3 ^$^	66 (7.2)	853 (92.8)	79.0 (60.0, 100.0)	89.0 (69.0, 108.0)	0.678 (0.439, 1.047)	32.2 (−4.7, 56.1)	0.611 (0.387, 0.967)	38.9 (3.3, 61.3)
Household contact setting								
Group 2-2^#^	18 (10.6)	152 (89.4)	250.0 (216.0, 266.0)	253.0 (246.3, 264.0)	Ref.	..	Ref.	..
Group 3-3 ^$^	44 (13.6)	280 (86.4)	78.0 (64.0, 94.0)	87.0 (72.5, 94.0)	1.327 (0.741, 2.377)	−32.7 (−137.7, 25.9)	1.600 (0.879, 2.911)	−60.0 (−191.1, 12.1)
Non-household contact setting								
Group 2-2^#^	52 (7.7)	624 (92.3)	242.0 (201.0, 260.0)	243.0 (207.8, 263.0)	Ref.	..	Ref.	..
Group 3-3 ^$^	100 (4.8)	1993 (95.2)	82.0 (60.0, 106.0)	88.0 (74.0, 113.0)	0.602 (0.426, 0.852)	39.8 (14.8, 57.4)	0.542 (0.377, 0.779)	45.8 (22.1, 62.3)

VE, vaccine effectiveness; * Variables adjusted in the model were sex of index cases and their close contacts, age of index cases and their close contacts, contact setting, and exposure to index cases at onset of indexes; ^#^ Fully vaccinated close contacts seeded by fully vaccinated index cases; ^$^ Booster-vaccinated close contacts seeded by booster-vaccinated index cases.

## Discussion

In this study, we conducted a retrospective cohort study to evaluate the effectiveness of booster vaccination of inactivated COVID-19 vaccines against being infected (susceptibility), infecting others (infectiousness), and spreading the COVID-19 among individuals (transmission) during an Omicron BA.2 sub-lineage outbreak in Shenzhen, China. Under the terms of the study, the stringent restriction measures were still active to prevent Omicron BA.2 infection and all the close contacts and their index cases were carefully traced and monitored. Consequently, we could assess the effectiveness of booster vaccination of inactivated COVID-19 vaccines against susceptibility, infectiousness, and transmission.

Several studies indicated that Omicron infection was less severe compared with previous SARS-CoV-2 waves, leading to a relatively low risk of COVID-19-related hospitalization and COVID-19-related death (39–42). However, this difference might be due to population natural immunity as well as COVID-19 vaccine-induced immunity. Therefore, researchers have great interest in estimating the VE against the Omicron different sub-lineages. An important issue is to evaluate the role of inactivated COVID-19 vaccine boosters against Omicron BA.2 infection caused by Omicron BA.2 sub-lineage.

In this study, our results indicated that booster vaccination of inactivated COVID-19 vaccines did not provide any protection against Omicron BA.2 infection. As far as we know, compared with full vaccination, only two published studies estimated the relative effectiveness of inactivated COVID-19 vaccine boosters against infection caused by Omicron BA.2 variant (35, 43). Consistent with our results, a test-negative case-control study in Guangzhou, China found that booster immunization of inactivated COVID-19 vaccines did not provide any protective effect against Omicron BA.2 infection (35). Another retrospective cohort study found that the inactivated COVID-19 vaccine boosters only provided low level of protection against Omicron BA.2 infection (43). Although the present study found that booster vaccination of inactivated COVID-19 vaccines cannot offer effective protection against susceptibility caused by Omicron BA.2 variant. However, several published studies (24, 30–34, 44) indicated that the importance of COVID-19 vaccine boosters to mitigate the severity of COVID-19, especially in old adults, which is consistent with WHO recommendation about administration of COVID-19 vaccines for this group (45). Moreover, several previous studies (24, 30–34, 44) and meta-analyses (13, 46, 47) confirmed that COVID-19 vaccine boosters only provided low level of protection against infection caused by Omicron variant. These results indicated that the inactivated COVID-19 vaccine boosters were inefficient in preventing Omicron BA.2 epidemic. However, published studies were not comprehensive enough because the VE measured in their studies was restricted to evaluating against susceptibility (infection).

Another important aim of this study is to estimate the effectiveness of inactivated COVID-19 vaccine boosters against infectiousness and transmission caused by Omicron BA.2 variant. We found that the overall effectiveness of booster vaccination of inactivated COVID-19 vaccines against infectiousness was 44.9% and indicating that booster vaccination can reduce the infectivity among the infected individuals for adults aged ≥ 18 years older. The reduced infectiousness among booster-vaccinated COVID-19 cases can be because vaccination shortens the duration of time of high transmission potential, minimizes symptom duration, and furthermore may restrict tissue dissemination of active virus (48–50). VE against transmission which refers to the ability of COVID-19 vaccine to block the virus transmission chain and cut off community transmission. Overall, the inactivated vaccine boosters only provided a low level of protection in preventing transmission. Our results indicated that the individuals who received the booster vaccination had a 29.0% decreased risk of spreading the Omicron BA.2 virus from one to another. Then, we conducted subgroups according to gender and contact setting. We found that the inactivated COVID-19 vaccines boosters provided effective protection against infectiousness and transmission for the female and non-household close contacts. Further analysis observed that females had a higher secondary infection rate (14.7%) than males (7.0%) among group 2-2, which may be attributed to females having more frequent household contact with COVID-19 cases. Previous studies have indicated that the household setting is the main route of transmission due to more frequent and longer unprotected exposure to SARS-CoV-2 (51, 52). However, it is needed to further confirm the differences of VE for gender.

Although we previously applied same data to evaluate the effectiveness of inactivated COVID-19 vaccine booster immunization against transmission in Shenzhen during a BA.2 outbreak period from 1 February to 21 April 2022 (53). However, we only considered the COVID-19 vaccination status of index cases. In this study, to further distinguishing the effectiveness of booster vaccination of inactivated COVID-19 vaccines, we applied four sub-cohorts according to the inactivated COVID-19 vaccination status of both the close contacts and their index cases to evaluate the effectiveness of inactivated vaccine booster immunization against susceptibility), infectiousness, and transmission. Therefore, the present study adds unique contributions to the scientific literature. First, it expanded on previous studies on the effectiveness of inactivated vaccine boosters only against infection (susceptibility). Second, it offered preliminary evidence of the effectiveness of inactivated vaccine boosters against susceptibility, infectiousness, and transmission caused by Omicron BA.2 subvariant using a cohort study design. Third, it was the first study to comprehensively assess the effectiveness of inactivated COVID-19 vaccine boosters against susceptibility, infectiousness, and transmission of Omicron BA.2 subvariant using contact tracing data. However, there were also several limitations. Firstly, this study did not assess the COVID-19-related hospitalization because all SARS-CoV-2 positive people must be isolated and hospitalized in China regardless of the severity of COVID-19 during the study period. Therefore, the current results cannot be extended to a more severe clinical range of COVID-19. Secondly, unmeasured exposure factors (e.g., conversation, shared room) might compromise the validity of our results although we had tried to control the known covariates. Thirdly, some behaviors (e.g., social distancing and personal protection) that also may affect our results. Fourthly, we did not consider mixing observations from different indexes and repeating observations from the same index due to data limitation in this study. Lastly, due to the short period of observation, we did not analyze the VE of inactivated COVID-19 vaccines from different vendors. Moreover, we also did not evaluate the duration of booster dose compared with full vaccination in this study.

In summary, booster vaccination of the inactivated COVID-19 vaccines provides low level of protection and moderate level of protection against Omicron BA.2 transmission and infectiousness, respectively. However, booster vaccination does not provide any protective effect against Omicron BA.2 susceptibility. These findings indicate that it is continuously needed assessing VE against the Omicron variant and may offer enough information to help develop COVID-19 vaccination strategies.

## Data availability statement

The original contributions presented in the study are included in the article/supplementary material. Further inquiries can be directed to the corresponding authors.

## Ethics statement

The studies involving humans were approved by China CDC Ethical Review Committee (approval number 202210). The studies were conducted in accordance with the local legislation and institutional requirements. Written informed consent for participation was not required from the participants or the participants’ legal guardians/next of kin in accordance with the national legislation and institutional requirements.

## Author contributions

YuL: Data curation, Formal analysis, Investigation, Methodology, Project administration, Validation, Writing – original draft, Writing – review & editing. JS: Formal analysis, Writing – original draft, Writing – review & editing. JZ: Formal analysis, Writing – original draft, Writing – review & editing. ZQ: Investigation, Writing – review & editing. ZA: Investigation, Writing – review & editing. WG: Data curation, Investigation, Writing – review & editing. JW: Data curation, Investigation, Writing – review & editing. YiL: Data curation, Investigation, Writing – review & editing. XZ: Conceptualization, Investigation, Project administration, Supervision, Writing – review & editing. XH: Conceptualization, Data curation, Project administration, Supervision, Writing – original draft, Writing – review & editing.
